# Translation to Portuguese and cross-cultural adaptation of the epilepsy transition readiness checklist for use in Brazil

**DOI:** 10.1016/j.clinsp.2024.100432

**Published:** 2024-07-15

**Authors:** Daniela Fontes Bezerra, Rudá Alessi, Danielle Molinari Andrade, Rubens Wajnsztejn, Marco Akerman

**Affiliations:** a Neurology Discipline, Faculdade de Medicina do ABC, Santo André, SP, Brazil; b Adult Genetic Epilepsy Program, Division of Neurology, Krembil Brain Institute, Toronto Western Hospital, University of Toronto, Toronto, Canada; c Department of Politics, Management and Health, Faculdade de Saúde Pública, Universidade de São Paulo, São Paulo, SP, Brazil

**Keywords:** Epilepsy, Readiness checklist, Translation, Cross-cultural adaptation, Transition, Child epilepsy

## Abstract

**Objectives:**

Planning for the child and adolescent to have a safe handling in the epilepsy transition process is essential. In this work, the authors translated the “Readiness Checklists” and applied them to a group of patients and their respective caregivers in the transition process to assess the possibility of using them as a monitoring and instructional instrument.

**Methods:**

The “Readiness Checklists” were applied to thirty adolescents with epilepsy and their caregivers. The original English version of this instrument underwent a process of translation and cultural adaptation by a translator with knowledge of English and epilepsy. Subsequently, it was carried out the back-translation and the Portuguese version was compared to the original, analyzing discrepancies, thus obtaining the final version for the Brazilian population.

**Results:**

Participants were able to answer the questions. In four questions there was an association between the teenagers' educational level and the response pattern to the questionnaires. The authors found a strong positive correlation between the responses of adolescents and caregivers (Rho_Spearman_ = 0.837; *p* < 0.001). The application of the questionnaire by the health team was feasible for all interviewed patients and their respective caregivers.

**Conclusion:**

The translation and application of the “Readiness Checklists” is feasible in Portuguese. Patients with lower educational levels felt less prepared for the transition than patients with higher educational levels, independently of age. Adolescents and caregivers showed similar perceptions regarding patients' abilities. The lists can be very useful tools to assess and plan the follow-up of the population of patients with epilepsy in the process of transition.

## Highlights

• An inefficient transition process for children and adolescents with epilepsy may compromise adherence to consultations, and treatment and increase the hospitalization rate.
• The “Readiness Checklists” were translated into Portuguese and applied to a group of patients and their respective caregivers in the transition process.
• The lists can be very useful tools to assess and plan the follow-up of the population of patients with epilepsy in the process of transition.

## Introduction

Epilepsy is a chronic brain disease caused by diverse etiologies and characterized by the recurrence of unprovoked epileptic seizures and their neurobiological, cognitive, psychological and social consequences.^1^ The incidence of epilepsy in the Western population is presumed to be 1 case per 2,000 people per year, being higher in the first year of life and increasing after 60 years of age, thus characterizing two incidence peaks. The lifetime prevalence of epilepsy is around 3 % to 5 %.^2^

Advances in pediatric medicine resulted in interventions that effectively treat conditions previously considered untreatable. In the last two decades, this scenario has brought specific needs to children with epilepsy and their families, because in addition to living with this chronic condition, patients go through the natural biological development of the childhood-pubertal transition.^3^ The introduction of a “transition” process aims at planning directed towards a context of medical, psychosocial, and educational/professional needs of adolescents and young people with chronic health conditions. This transition step must be an active process that addresses the overall needs of adolescents who will be transferred from pediatric to adult treatment centers. Plan and managing multiple variables such as frequent appointments and collection of laboratory tests, adherence to the use of medications, changes in lifestyle including self-care, and identification of stressors and crisis triggers such as avoiding sleep deprivation, among others, increases the complexity of this process.^3^

There are 65 million people living with epilepsy in the world and approximately 10.5 million of these patients are children.^4^ Therefore, the transition from pediatric patients with epilepsy to adulthood is challenging for health systems, as well as for many young people with epilepsy and their families. The Ministry of Health and Long-Term Care of the Province of Ontario, Canada, created a transition working group to develop recommendations for the transition process for patients with epilepsy.^5^ Among the main conclusions of this work, it was observed that the early identification of adolescents at risk of poor transition is essential. The study proposes seven steps that can facilitate the transition, promoting uninterrupted and adequate care for young people with epilepsy leaving the pediatric universe. The third step suggested by the working group is to “Determine Transition Readiness of Patients and their Parents”. To this end, “Readiness Checklists” have been proposed to help assess and improve patients' understanding of their health condition, thus facilitating the transition process.

An efficient transition process for children and adolescents with epilepsy is essential, since if the process is carried out improperly, it may compromise adherence to consultations, and treatment and increase the hospitalization rate, including intensive care.^6^ There are few studies that address the different aspects of the transition process of patients with epilepsy such as long-term planning, which includes the development of strategies that address the specificities of the age group, recognition of risk factors, and parental involvement as relevant factors. In Brazil, there is no study with this profile carried out in a population of a tertiary epilepsy center. In this work, the authors translated the “Readiness Checklists” and applied them to a group of 30 patients and their respective caregivers in the transition process to assess the possibility of using them as a monitoring and instructional instrument.

## Methods

### Study design

A convenience sample consisting of adolescents and their caregivers was eligible for this cross-sectional study, following the STROBE Statement. The adolescents were regularly monitored at the Epilepsy Transition Outpatient Clinic of the Faculdade de Medicina do ABC and were included in the study from March 2022 to April 2023, complying with the following inclusion criteria: age between 12 and 18 years old, normal intellectual capacity or mild Intellectual Disability (ID), presenting a well-defined diagnosis of epilepsy, according to criteria from the International League Against Epilepsy (ILAE).^7^ The respective caregivers, responsible most of the time for the patient and at least 18 years old, were also included in the study. Adolescents and caregivers with moderate or severe mental disabilities were excluded. Selected adolescents and their respective caregivers underwent anamnesis to characterize the clinical history. The study included an interview to obtain demographic data and answers to questions from the lists of patients and their caregivers. The original English version of this instrument^5^ underwent a process of translation and cultural adaptation by a translator with knowledge of English and epilepsy. Subsequently, an independent and native English teacher carried out the back-translation, and the back-translated and Portuguese versions were compared to the original, analyzing discrepancies in terms of concepts and content, thus obtaining the final version for the Brazilian population (Table 1, Table 2). The questionnaire was applied to patients and caregivers through an interview conducted by trained physicians linked to the service.

**Table 1:** Epilepsy transition readiness checklist for teenager (Portuguese and English versions).

**For each of the following statements please select the response that best suits you:**	No, I do not know	No, but I am learning to do this	Yes, I have started doing this	Yes, I always do this	Does not apply to me
**Para cada afirmação por favor selecione a resposta que melhor se aplica a você:**	Não, eu não sei	Não, mas eu estou aprendendo a fazer isso	Sim, eu comecei a fazer isso	Sim, eu sempre faço isso	Não se aplica para mim
**Question**	**Portuguese version**			**English version**	

3.1	Eu posso descrever minha condição de saúde e explicar os meus cuidados a outros.			I can describe my health condition and explain my health care needs to others.	
3.2	Eu sei o que desencadeia minhas crises e como minimizar gatilhos.			I know what triggers my seizures and how to minimize the triggers.	
3.3	Eu sei o que fazer em caso emergência relacionada a minha condição de saúde.			I know what to do in the event of a medical emergency relating to my condition (first aid; when to call 911)	
3.4	Eu sei comunicar o meu médico sobre mudanças incomuns na minha saúde (por exemplo: efeitos colaterais de medicamentos).			I know how to call the doctor about unusual changes in my health (for example: medication side effects)	
3.5	Eu sei os nomes dos medicamentos que tomo.			I know the names of the medications I take	
3.6	Eu sei como tomar os medicamentos corretamente por conta própria e ter um meio de não esquecer.			I know how to take medications correctly on my own and have a system in place to remind me when to take them.	
3.7	Eu sei quando os meus medicamentos irão acabar.			I know when and how to reorder medications before they run out.	
3.8	Eu tive uma conversa sobre como certos medicamentos podem ter impacto em possíveis gestações/ gravidez.			I have had a discussion about how certain medications can impact birth control and pregnancy.	
3.9	Eu faço uma lista de perguntas para perguntar ao meu médico antes de ir na consulta.			I make a list of questions to ask my doctor before going to appointments (appointments, medications, seizures, etc.)	
3.10	Eu organizo e acompanho minhas informações de saúde (consultas, medicamentos, convulsões etc.).			I make a list of questions to ask my doctor before going to appointments.	
3.11	Eu posso chegar nas consultas médicas por conta própria.			I can get to medical appointments on my own.	
3.12	Eu passo um tempo sozinho com meu médico em cada consulta.			I spend time alone with my health care provider at each appointments.	
3.13	Eu falo por mim e digo aos outros o que preciso durante os cuidados de saúde/da minha saúde.			I speak up for myself and tell others what I need during health care visits.	
3.14	Eu tenho discutido sexualidade com a equipe de saúde (DSTs/contracepção) sobre doenças sexualmente transmissíveis e também sobre como evitar uma gestação.			I have discussed sexuality and reproductive health with my health care team (consent/sexually transmitted infections/contraception).	
3.15	Eu sei como meu estilo de vida pode afetar minha condição de saúde (por exemplo, uso de álcool, drogas, falta de sono...)			I know how my lifestyle can impact my health condition and how to discuss this with my health care team (e.g., use of alcohol, drugs, lack of sleep, etc.).	
3.16	Eu entendo as regras e regulamentos sobre epilepsia e dirigir.			I understand the rules and regulations about epilepsy and driving.	
3.17	Eu entendo as implicações da minha condição de saúde na minha vida em relação a carreira a ser escolhida e futuro emprego.			I understand the implications of my heath condition on career choice and future employment.	
3.18	Eu conheço meus direitos legais como uma pessoa que vive com essa condição de saúde e como acessar as acomodações necessárias na escola e em trabalhos.			I know my legal rights as a person living with this health condition and how to access necessary accommodations at school and at work.	
3.19	Eu sei do meu direito à privacidade, confidencialidade e tomada de decisões sobre minha saúde.			I know about my right to privacy, confidentiality, and decision making regarding my health.	
3.20	Eu sei como divulgar/contar sobre a minha epilepsia para amigos, colegas, colegas de trabalho e outros.			If I chose to, I know how to disclose my epilepsy to friends, classmates, coworkers, and others.	
3.21	Eu sei como acessar/o que fazer e a quem procurar/ os suportes que preciso se me sentir estressado, deprimido ou ansioso.			I know how to access the supports I need if I feel stressed, depressed, or anxious.	
3.22	Eu sei o que esperar em serviços para adultos e como isso é diferente dos serviços pediátricos (para crianças e adolescentes).			I know what to expect in adult services and how it differs from pediatric services.

**Table 2:** Epilepsy transition readiness checklist for caregivers (Portuguese and English versions).

**For each of the following statements please select the response that best suits you:**	No, my child does not know this	No, but my child is learning to do this	Yes, my child has started doing this	Yes, my child always does this	Does not apply to my child
**Para cada afirmação por favor selecione a resposta que melhor se aplica a você:**	Não, meu filho não sabe disso	Não, mas meu filho está aprendendo a fazer isso	Sim, meu filho começou a fazer isso	Sim, meu filho sempre faz isso	Não se aplica para meu filho
**Question**	**Portuguese version**			**English version**	

4.1	Meu filho tem uma compreensão da sua condição de saúde e como está sendo administrada (tipo de convulsões, quando uma convulsão é uma emergência médica, primeiros socorros, tratamento, etc.).			My child has an understanding of his or her health condition and how it is being managed (type of seizures, when a seizure is a medical emergency, first aid, treatment, etc.).	
4.2	Meu filho pode descrever sua saúde e condições para os outros (médico, emergência pessoal, escola, empregador, etc.).			My child can describe his or her health condition to others (physician/emergency personnel, school, employer, etc.).	
4.3	Meu filho participa de cuidados de saúde e discussões sobre ele ou ela.			My child takes part in health care discussions about him or herself.	
4.4	Meu filho organiza e acompanha a sua própria informação de saúde (consultas, medicamentos, convulsões, resultado de exames).			My child organizes and keeps track of his/her own health information (appointments, medications, seizures, test results).	
4.5	Meu filho sabe como conseguir consultas.			My child knows how to get him/herself to health care appointments.	
4.6	Meu filho tem um plano para quando ele se sente estressado, deprimido ou ansioso.			My child has a plan in place for when he feels stressed, depressed, or anxious.	
4.7	Meu filho sabe o que a sua condição de saúde pode afetar no futuro (por exemplo: prognóstico, casamento, filhos).			My child knows what his/her health condition can bring in the future (e.g. prognosis, marriage, children).	
4.8	Meu filho fala por si e consegue sozinho cuidar da sua saúde.			My child speaks up for him/herself and spends some time alone with health care provider at each visit (when necessary).	
4.9	Meu filho conversa com os profissionais de saúde sobre como sua condição é afetada por tabaco, álcool e outras drogas.			My child talks to health care providers about how his/her condition is affected by tobacco, alcohol, and other drugs.	
4.10	Meu filho fala com os profissionais de saúde sobre saúde sexual e reprodutiva e questões como contracepção e DSTs.			My child talks to health care providers about sexual and reproductive health issues (contraception, sexually transmitted infections, consent).	
4.11	Meu filho tem uma rede de amigos, família ou outras pessoas na comunidade, como suporte que podem apoiá-lo em momentos de estresse.			My child has a network of friends, family, or other community supports that can support him/her in times of stress.	
4.12	Meu filho está ciente das carreiras/empregos que podem não ser adequados para uma pessoa que vive com epilepsia			My child is aware of careers that may not be suitable for a person living with epilepsy.	
4.13	Meu filho está ciente dos regulamentos em torno de dirigir/condução e epilepsia			My child is aware of the regulations around driving and epilepsy.	
**For each of the following statements please select the response that best suits you:**	No, I do not know	I know some of this	I know most of this	I know all about this	Does not apply

**Para cada afirmação por favor selecione a resposta que melhor se aplica a você:**	Não, eu não sei	Eu sei parte disso	Eu sei a maior parte disso	Eu sei tudo sobre isso	Não se aplica

4.14	Entendo o direito de meu filho à confidencialidade e o direito de consentimento informado.			I understand my child's right to confidentiality and the right to informed consent	
4.15	Estou ciente dos recursos da comunidade que pode me ajudar no processo de transição.			I am aware of community resources that can assist me with the transition process	
4.16	Eu estou trabalhando com meu filho em um plano de transição.			I am working with my child on a transition plan	
4.17	Eu falo com meu filho sobre planejamento de carreira e como sua condição de saúde pode impactar.			I speak with my child about career life planning and how his/her health condition can impact thisemployment.	

### Ethics

The study was approved and conducted following institutional review board regulations of the Research Ethics Committee of the Faculdade de Medicina do ABC, São Paulo, Brazil (Protocol n° CAAE 53143820.8.0000.0082). All participants signed a consent form prior to any study procedure.

### Statistical analysis

All analyses were performed using the Jamovi software (Version 2.4).^8^^,^^9^ The distribution of all continuous and categorical variables was analyzed with nonparametric statistics. Fisher's exact test was applied to verify the association between the patients' and caregivers’ educational level and the response pattern to the questionnaires. The effect of patients' and caregivers’ ages on the pattern of answers to the questionnaire was evaluated by the Kruskal-Wallis test. Spearman's correlation coefficients were used to measure the correlation between teenagers' and caregiver's answers. Therefore, numbers from 0 to 4 were assigned to the responses of adolescents and caregivers regarding the inventory of skills. For all analyses, it was assumed a significance level of 0.05.

## Results

**Fig. 1:**
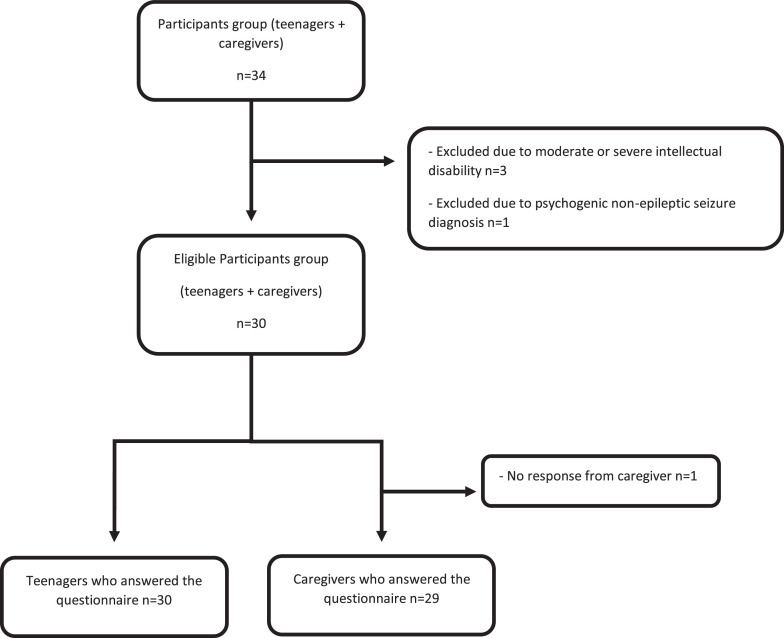
Experimental design and number of patients included in the study.

Thirty-four participants were recruited. Three adolescents were excluded due to moderate or severe intellectual disability and one due to a diagnosis of psychogenic non-epileptic seizure. Thirty participants were included in the study. The experimental design and number of patients included in the study can be seen in Fig. 1. Their mean age was 15.1 ± 1.80 years, ranging from 12 to 18 years. Most patients are female (56.7 %) and 14 patients (46.7 %) have secondary education (Table 3). When asked if they knew what epilepsy is, half (50 %) answered “yes”. Table 4 refers to the characteristics of caregivers who answered the questionnaire. The majority (51.7 %) are between 35 and 44 years old, female (79.3 %) and have completed or incomplete secondary education (55.2 %). 78.6 % of participants have a family income ranging from BRL 1301.00 to BRL 5200.00, which corresponds to 1 to 4 minimum wages. 31 % of caregivers reported that the patient is currently free of seizures and 12 patients (41.4 %) have anxiety in addition to epilepsy.

**Table 3:** Demografic characteristics of the sample patients (*n* = 30).

	N	n (%)	Mean ± SD	Min‒Max
**Age (years)**	**29**		15 ± 1.8	12‒18
**Gender**	**30**			
Male		13 (43.3)		
Female		17 (56.7)		
**Education**	**30**			
Primary education		12 (40)		
Secondary education		14 (46.7)		
Higher education or Supplementary		2 (6.7)		
Technical course		1 (3.3)		
None		1 (3.3)		
**Do you know what epilepsy is?**	**30**			
No		15 (50)		
Yes		15 (50)

**Table 4:** Demografic characteristics of the sample caregivers (*n* = 29).

	N	n (%)
**Age (years)**	**29**	
18‒24		1 (3.4)
35‒44		15 (51.7)
45‒54		11 (37.9)
55‒64		2 (6.9)
**Sex**	**29**	
Male		23 (79.3)
Female		6 (20.7)
**Education**	**29**	
Primary education		3 (10.3)
Secundary education		16 (55.2)
Technical course		4 (13.8)
Higher education		3 (10.3)
Postgraduate studies		2 (6.9)
**Family income**	**28**	
From zero to BRL 260.00 (0‒0.2 MW)		1 (3.6)
From BRL 781.00 to BRL 1300.00 (0.6‒1 MW)		2 (7.1)
From BRL 1301.00 to BRL 2600.00 (1‒2 MW)		12 (42.9)
From BRL 2601.00 to BRL 5200.00 (2‒4 MW)		10 (35.7)
From BRL 5200.00 to BRL 7800.00 (4‒6 MW)		3 (10.7)
**Marital status**	**29**	
Single		5 (17.2)
Married/Living as married		16 (55.2)
Divorced		8 (27.6)
**Frequency of patient seizures**	**29**	
Crisis free		9 (31.0)
1 × a year		7 (24.1)
Every 6-months		5 (17.2)
Every 3-months		3 (10.3)
Monthly		1 (3.4)
Weekly		1 (3.4)
More than 2 × a week		3 (10.3)
**Associated diseases**^a^	**29**	
Depression		2 (6.9)
Anxiety		12 (41.4)
ADHD		9 (31.0)

ADHD, Attention-Deficit/Hyperactivity Disorder. MW, Minimum Wage.

a The same patient may have more than one associated disease.

Regarding the responses to the questionnaires, all patients were able to answer the questions presented, with the exception of one patient who did not present age data. One of the companions did not feel able to answer, since she was the patient's neighbor and not a direct caregiver. Regarding the questionnaire applied to the patients, referring to the skills inventory, the possible answers were: No, I don't know; No, but I'm learning to do this; Yes, I started doing that; Yes, I always do that and It does not apply to me. Patients were able to answer all questions presented (Table 5). Fisher's exact test was performed to verify whether there is any association between the patients' educational level and the response pattern to the questionnaires. The authors verified that in only four questions of the questionnaire (Question 3.6: *p* = 0.033; Question 3.9: *p* = 0.05; Question 3.12: *p* = 0.038 and Question 3.22: *p* = 0.025) this association occurred and patients with lower education level (primary education) answered more frequently than “No, I don't know”; and “No, but I'm learning to do this”; while patients with secondary education, higher education or technical course answered more frequently “Yes, I started doing that” and “Yes, I always do that”. The age of the patients had no effect on the pattern of answers to the questionnaire, using the Kruskal-Wallis test.

**Table 5:** Teenagers’ responses to epilepsy transition readiness checklist for teenager (*n* = 30).

Question	N	No, I do not know		No, but I am learning to do this		Yes, I have started doing this		Yes, I always do this		Does not apply to me	
		**n**	**%**	**n**	**%**	**n**	**%**	**n**	**%**	**n**	**%**
**3.1**	30	11	36.7	8	26.7	5	16.7	6	20	0	0
**3.2**	30	12	40	4	13.3	9	30	4	13.3	1	3.3
**3.3**	30	15	50	3	10	9	30	3	10	0	0
**3.4**	30	13	43.3	5	16.7	6	20	6	20	0	0
**3.5**	30	9	30	3	10	7	23.3	11	36.7	0	0
**3.6**	30	7	23.3	7	23.3	5	16.7	11	36.7	0	0
**3.7**	30	11	36.7	5	16.7	6	20	8	26.7	0	0
**3.8**	30	16	53.3	3	10	2	6.7	4	13.3	5	16.7
**3.9**	29	16	55.2	9	31	3	10.3	0	0	1	3.4
**3.10**	30	22	73.3	5	16.7	3	10	0	0	0	0
**3.11**	30	21	70	5	16.7	4	13.3	0	0	0	0
**3.12**	30	8	26.7	8	26.7	11	36.7	3	10	0	0
**3.13**	30	7	23.3	15	50	6	20	2	6.7	0	0
**3.14**	30	21	70	2	6.7	1	3.3	3	10	3	10
**3.15**	30	8	26.7	12	40	8	26.7	2	6.7	0	0
**3.16**	30	19	63.3	3	10	3	10	0	0	5	16.7
**3.17**	30	24	80	2	6.7	2	6.7	2	6.7	0	0
**3.18**	30	25	83.3	2	6.7	0	0	2	6.7	1	3.3
**3.19**	30	23	76.7	4	13.3	2	6.7	1	3.3	0	0
**3.20**	30	10	33.3	11	36.7	5	16.7	4	13.3	0	0
**3.21**	30	12	40	11	36.7	4	13.3	2	6.7	1	3.3
**3.22**	30	22	73.3	6	20	2	6.7	0	0	0	0

Caregivers were instructed to answer the questionnaire related to the inventory of patients' skills in the caregiver's view and the possible answers to the questions were: No, my son doesn't know that; No, but my son is learning to do this; Yes, my son started doing this; Yes, my son always does this, and it does not apply to my son (Table 6). The authors did not find an association between the education level and the caregivers' responses, nor did the age interfere with the caregivers' response pattern.

**Table 6:** Caregivers’ responses to epilepsy transition readiness checklist for caregivers (*n* = 29).

Question	N	No, my child does not know this		No, but my child is learning to do this		Yes, my child has started doing this		Yes, my child always does this		Does not apply to my child	
		**n**	**%**	**n**	**%**	**n**	**%**	**n**	**%**	**n**	**%**
**4.1**	29	9	31	7	24.1	7	24.1	6	20.7	0	0
**4.2**	29	9	31	5	17.2	5	17.2	10	34.5	0	0
**4.3**	27	10	37	6	22.2	3	11.1	8	29.6	0	0
**4.4**	29	13	44.8	8	27.6	4	13.8	4	13.8	0	0
**4.5**	29	21	72.4	5	17.2	2	6.9	1	3.4	0	0
**4.6**	29	16	55.2	7	24.1	2	6.9	4	13.8	0	0
**4.7**	29	15	51.7	4	13.8	6	20.7	4	13.8	0	0
**4.8**	29	15	51.7	5	17.2	6	20.7	3	10.3	0	0
**4.9**	29	13	44.8	3	10.3	5	17.2	8	27.6	0	0
**4.10**	29	22	75.9	1	3.4	3	10.3	3	10.3	0	0
**4.11**	29	5	17.2	11	37.9	9	31	4	13.8	0	0
**4.12**	29	21	72.4	4	13.8	0	0	4	13.8	0	0
**4.13**	29	9	31	12	41.4	4	13.8	4	13.8	0	0

**Question**	**N**	**No, I do not know**		**I know some of this**		**I know most of this**		**I know all about this**		**Does not apply**	
		**n**	**%**	**n**	**%**	**n**	**%**	**n**	**%**	**n**	**%**
**4.14**	28	7	25	14	50	3	10.7	4	14.3	0	0
**4.15**	29	3	10.3	16	55.2	6	20.7	4	13.8	0	0
**4.16**	29	4	13.8	14	48.3	7	24.1	4	13.8	0	0
**4.17**	29	19	65.5	4	13.8	2	6.9	4	13.8	0	0

Numbers from 0 to 4 were assigned to the responses of adolescents and caregivers regarding the inventory of skills, with 0 being the response “Does not apply to me” and “Does not apply to my child”, 1 representing the response “No, I don't know ” and “No, my son does not know that”; 2, “No, but I am learning to do this” and “No, but my son is learning to do this”; 3, “Yes, I started doing that” and “Yes, my son started doing that” and 4, “Yes, I always do that” and “Yes, my son always does that”. The values of the responses of each participant were added and a linear correlation analysis was performed between the responses of adolescents and caregivers. For this analysis, the authors removed data from the patient whose companion did not respond to the questionnaire. The authors found a strong positive correlation between the responses of adolescents and caregivers (Rho _Spearman_ = 0.837; *p* < 0.001).

## Discussion

Epilepsy is the most common chronic disorder of childhood; remission of epileptic seizures occurs in around 50 % of patients and the rest become adults living with epilepsy.^10^^,^^11^ Therefore, a transfer to the service specialized in adults with chronic epilepsy, or ideally a transition, would be of paramount importance.^12^ The direct neurological consequences of epilepsy have an impact on autonomy, self-care, and family dynamics, and can promote risk factors; planning for children and adolescents to have a safe handling of the transition process is essential for patients to live in a healthy way with epilepsy in adulthood. There is growing knowledge about the impact of epilepsy in this age group and the possible positive gains in relation to the transition process; as a result, some centers are modifying the generic programs, used for several chronic diseases, to include specific modules for epilepsy.^6^

In this work, the authors performed the translation and application of the “Readiness Checklists”, developed by a working group created by the Ministry of Health and Long-Term Care of the Province of Ontario, for a group of 30 patients and their respective caregivers in the process of transition. The working group created to develop recommendations for the transition process was composed of a multidisciplinary team and addressed topics such as the diagnosis and management of seizures, mental health and psychosocial needs, and financial aspects.^5^

The authors verified that the application of the questionnaire by the health team and resident physicians under the supervision of the coordinating physician was feasible for all interviewed patients and their respective caregivers. Despite the small sample size, it was possible to verify that the cultural adaptation of the questionnaire was quite satisfactory. However, in order to obtain more adequate planning and better management of the transition process, it will be important to apply the questionnaire to a larger number of patients. For the vast majority of questions, the authors did not observe an association between education level and age in the pattern of responses to the questionnaire, both for patients and caregivers.

A systematic review with quality criteria established by the Effective Practice and Organization of Care group,^13^ highlighted the relevance of the transition of adolescents with chronic diseases from pediatric services to adult services. Generic transition programs, which promote the preparation of children and adolescents with chronic diseases in general, for adult services with chronic diseases, must have nine elements for planning the transition process: starting age, contact with the adult service team before the end of the process, promotion of autonomy, a transition plan, parental involvement, cohesive health team, skills training respecting the patient's lifestyle, professional reference for each patient, and the presence of a coordinator for the transition team.^14^ A prospective study with diabetic patients with cerebral palsy and autism spectrum disorder showed strong evidence that three of the nine elements are associated with a favorable outcome at the end of the transition: adequate parental involvement, promotion of autonomy, and contact with the care team adult service before finishing the process.^15^

The objectives to be achieved with the transition program can be elucidated in three points. The first point is to provide young people with epilepsy with appropriate education regarding their condition, such as the type of epilepsy, clinical course and treatment. This leads to an expectation on the part of the professional assistant that the youth will learn to take responsibility for themselves and learn to do things that successful adults with epilepsy need to do. Among these skills, the authors highlight adhering to medication, explaining the disease to their partner or caregiver, taking notes on the best way to conduct care for the disease, clearly discussing the problematic issues for him and being able to perceive and react to the difficulties that the health system in which he accompanies faces. The second point to be addressed would be in relation to the lifestyle of an adult with epilepsy, addressing aspects such as driving a car, the using of alcohol, and other substances. The third point would be to establish a growing bond of empathy with the neurologist who will promote care in adulthood and, not least, provide parental guidance and support so that parents and caregivers are also transitioned.^16^

It is recommended that patients, caregivers and healthcare staff use the information in the checklists as an instant reference to relevant medical information and should be used whenever the teenager visits a new doctor or emergency room. These portable health summaries increase patients' knowledge of their condition, improve their self-efficacy, and enhance collaboration between different healthcare assistants who will participate in the patient's care during the transition.^5^ At this first moment, the questionnaire was applied in the first consultation. The health service will carry out psychoeducational measures regarding autonomy and self-care during the infant-pubertal transition period. The questionnaire will be reapplied periodically, reassessing and comparing the answers when the patients turn 16‒17 years old, the moment of the first consultation with the adult neurology team. The comparison between the application phases of the questionnaire will be essential to infer whether the applied psychoeducational measures were effective.

## Conclusion

The translation of the “Readiness Checklists” prepared by the Transition Working Group of the Ministry of Health and Long-Term Care of the Province of Ontario, Canada, was completed and patients and caregivers were able to respond to all questions. The application of the list is feasible in Portuguese, with no association between education level and age in the pattern of responses to the questionnaire, both for patients and caregivers. The authors found a strong positive correlation between the responses of adolescents and caregivers, indicating that adolescents and guardians have similar perceptions regarding the patient's ability. The “Readiness Checklists” can be useful instruments to evaluate and plan the follow-up of the population of patients with epilepsy in the transition process.

## Authors’ contributions

DFB, RA and MA conceived and designed the study. DFB acquired the data and did the statistical analysis. DFB drafted the manuscript with critical input from RA, DA, RW and MA. All authors approved the final version.

## Declaration of competing interest

DFB, RA, DA, RW and MA have no conflicts to declare. The authors confirm that we have read the position of the Journal regarding ethical publication and declare that this manuscript is consistent with those guidelines.
